# Associations between organised sport participation and classroom behaviour outcomes among primary school-aged children

**DOI:** 10.1371/journal.pone.0209354

**Published:** 2019-01-02

**Authors:** Amanda Watson, Anna Timperio, Helen Brown, Trina Hinkley, Kylie D. Hesketh

**Affiliations:** Institute for Physical Activity and Nutrition (IPAN), School of Exercise and Nutrition Science, Deakin University, Geelong, Australia; University of New Mexico, UNITED STATES

## Abstract

**Introduction:**

Physical activity is positively associated with children’s classroom behaviour. However, less is known about how different types of physical activity contribute to these outcomes. This study examines associations between sport participation and classroom behaviour among primary school-aged children.

**Methods:**

Parents of 568 children aged 9–11 years reported child sport participation and classroom behaviour outcomes (school functioning, inattention, classroom behaviour (fidgetiness), acting without thinking and poor concentration). Sport participation included: duration (hours/week) and type (individual; team; team and individual). Regression analyses assessed associations between sport participation and classroom behaviour outcomes. Analyses adjusted for maternal education, and objectively-measured overall physical activity, and accounted for clustering by recruitment centre. Sex differences in associations were explored as a secondary aim.

**Results:**

In comparison to children who did not participate in sport, children who participated in sport displayed less inattention/hyperactivity (individual sport: *B* = -1.00;95%CI:-1.90, -0.00; team sport:-0.88;95%CI:-1.73, -0.03) and less acting without thinking (individual sport: OR = 0.35;95%CI:0.13,0.98), after adjusting for overall physical activity. There were no sex differences in associations.

**Conclusions:**

Findings indicated sport participation, one form of physical activity, was associated with less inattention/hyperactivity and acting without thinking, over and above the influence of overall physical activity levels. Parents may consider sport as one way to contribute to their child’s overall physical activity levels, although the impact of organised sport on classroom behaviour is modest at best.

## Introduction

In addition to the well-established physical and mental health benefits of physical activity, [1] emerging evidence from meta-analyses and systematic reviews show that physical activity may also be important for improving classroom behaviour [2, 3]. It is possible that physical activity improves cognitive function through psychosocial mechanisms, including self-esteem and physical self-perceptions [4]. Through improvements to cognitive function (e.g. attention and executive function) improvements in classroom behaviour may occur [5]. Classroom behaviour has been shown to predict later academic achievement (e.g. test scores), i.e. children who displayed more on-task classroom behaviour in preschool scored better in subsequent reading and spelling tests in years 1, 2 and 4 [6]. Thus, physical activity may not only benefit health, but may also benefit academic achievement via improvements in classroom behaviour and cognitive function. This study focusses on associations with classroom behaviour. While recently there has been increased interest in the impact of physical activity on classroom behaviour outcomes, less is known about whether particular types of physical activity are differentially associated with this outcome.

Organised sport is a specific type of physical activity and is typically defined as ‘organised, usually competitive, and can be played with a team or as an individual’ [7] Sport participation provides one way in which children can accrue physical activity. Organised sport is popular among children in Australia, with two thirds of children aged 5 to 14 years reporting participation in the previous 12 months [8]. However, participation rates decline with age, particularly in adolescence. For example, in Australia approximately 84 to 89% of primary and 41 to 75% of secondary school-aged children participated in organised sport in the previous week [8], contributing to the age related decline in physical activity. Thus, maintaining sport participation throughout childhood may be important for attaining recommended physical activity levels and associated health benefits.

In addition to health benefits, sport participation has been shown to be associated with many psychological and social benefits, beyond that derived from physical activity, including improvements to self-control and emotional regulation [7]. These distinct benefits are perhaps due to unique characteristics of sport participation, compared with other forms of physical activity. For example, compared with other more unstructured forms of physical activity (e.g. free play), when playing sport (e.g. tennis, football etc.), players are constantly thinking (e.g. thinking quickly and strategically about the rules, their next move, and their team mates actions) [9], which requires a great deal of focus. The focus practiced on the sports field may translate to better focus in the classroom.

Sport can be played as a team or as an individual. It is possible that these different types of sport may contribute differently to focus on the field, and subsequently focus in the classroom. Studies of the associations between sport participation and classroom behaviour among primary school-aged children are lacking [10]. One study reported that Taiwanese boys aged 6 to 12 years with attention deficit hyperactivity disorder displayed less parent reported attention problems following a 12-week table tennis (individual sport) intervention [11]. It has been suggested that individual sports require a greater level of focus than team sports as players don’t have their team mates to rely on and cannot “tune out” easily [12]. As such, children with attention deficit hyperactivity disorder are often encouraged to play individual sports [12]. Thus it is possible that individual sports may be more strongly associated with classroom behaviour, compared with team sports. However, another study of longitudinal associations between type of sport participation and school functioning among primary school-aged children showed school functioning scores were higher among those who participated in both team and individual sport, or in team sport alone, compared with individual sport alone [13]. A further study also found that team sport participation was associated with better classroom engagement, while individual sport participation was not [14]. With few studies and divergent results, to increase our understanding of associations between sport participation and classroom behaviour outcomes, associations with type of sport may be an important consideration.

Level of sport involvement (e.g. frequency and duration) may also influence the association with classroom behaviour outcomes, perhaps due to the extra physical activity. However, no studies among primary school-aged children have considered associations between levels of sport involvement with classroom behaviour outcomes. Previous studies among secondary school-aged children have investigated longitudinal associations between sport participation and academic achievement [15]. Results demonstrated that frequency of sports participation predicted English and Mathematics achievement at one year follow up [15].

Two previous studies among primary school-aged children considered associations between different levels of involvement in sport and academic achievement [16, 17]. Saevarsson and colleagues [17] investigated cross-sectional associations between frequency of sport participation and academic achievement among 248 children aged 9 years. Results indicated children who participated in sport ≤ once per week and 2–3 times per week had significantly lower mathematics scores, compared with children who participated in sport ≥ 4 times per week [17]. In contrast, Haapala and colleagues [16] concluded there was no association between duration of sport participation (minutes/day) in Grade 1 and academic achievement in Grades 1 to 3. That study also showed there were no sex differences in associations. However, no other study among primary school-aged children has investigated sex differences in associations between sport participation and classroom behaviour or academic outcomes. Due to known sex differences in classroom behaviour outcomes [18] as well as sport preferences [19] it may be important to investigate potential sex differences in associations.

While results are promising, no study among primary school aged children has adjusted for overall physical activity [13]. Thus, it is unclear whether improvements in classroom behaviour observed was due to sport participation per se, or the physical activity derived from sport participation. A study among high school students indicated that sport participation was associated with higher grade point average independent of overall physical activity [20]. This suggests that sport participation may have academic benefits beyond that derived from physical activity alone.

The current paper aims to expand on findings from previous studies by examining associations between (1) type (individual vs. team based) and duration (hours/week) of sport participation and classroom behaviour outcomes among primary school-aged children, and (2) whether or not associations persist after adjusting for overall physical activity. Sex differences in associations were explored as a secondary aim.

## Methods

### Participants and recruitment

Participants for this study were drawn from the third wave of the Healthy Active Preschool and Primary Years (HAPPY) study, based in metropolitan Melbourne, Australia. The HAPPY study is a cohort study focusing on children’s physical activity [21]. At baseline (2008–2009), a total of 71 (46%) childcare centres and 65 (47%) preschools agreed to be involved in the study. Parents (n = 9794) of children aged 3–5 years at each participating centre were invited to take part. To be eligible, children needed to be aged between 3 and 5 years, attending a participating centre. Of the 1032 families (10.5%) who consented at baseline, 766 agreed to be re-contacted in the future and formed the longitudinal arm of the study. Data for this paper is drawn from the third wave (T3) of the study, conducted in 2014–2016, in which 568 children (74% retention) aged 9 to 11 years took part.

Ethical approval for the HAPPY study was obtained from the Deakin University Human Research Ethics Committee (EC 291–2007), Department of Education and Early Childhood Development (2011_001008), and the Catholic Education Office (1714). Written informed consent was obtained from parents at each time point.

### Independent variable

Parents completed a survey at T3 in which they reported their child’s participation in organised sport in the past month: *“Thinking about the last month*, *has your child participated in any organised sports*?*”* Organised sport refers to a specific type of physical activity that is ‘organised, usually competitive, and can be played with a team or as an individual’ [7], and performed outside of school hours. Parents reported the names of up to six organised sports their child participated in over the past month, as well as the number of times per week and the total time (hours and minutes) their child participated in each sport. Responses were converted to overall duration of sport participation (hours/week), and also manually classified as team or individual, and participants were further categorised as (1) participates in team sport only (e.g. basketball, football, etc.); (2) participates in individual sport only (e.g. gymnastics, tennis, etc.); (3) participates in both team and individual sport; and (4) does not participate in sport. The definition used for team sports and individual sports were adopted from that used in a similar study [13]. Responses totalling greater than 14 hours per week were truncated to reflect realistic levels of participation.

### Dependent variables

#### School functioning

School functioning was measured via five items comprising the School Functioning subscale of the Pediatric Quality of Life (PedsQL) inventory [22]. Using the following question: *“In the past one month*, *how much of a problem has your child had with school functioning*?*”* parents reported their child’s school functioning across five domains: paying attention in class; forgetting things; keeping up with schoolwork; missing school because of not feeling well; and missing school to go to the doctor or hospital. The following codes were assigned: ‘never’ (score = 100); ‘almost never’ (score = 75); ‘sometimes’ (score = 50); ‘often’ (score = 25); ‘almost always’ (score = 0) [22]. A mean score was computed. The parent report version of the PedsQL school functioning subscale has a high level of internal consistency in parents of 8 to 12 year old children (r = 0.76), and was significantly correlated with academic achievement scores based on the Stanford 9 (r = 0.25, p<0.001) [23]. Further, both parent- and child- reported school functioning scores on the PedsQL have been shown to be similar [24]

#### Inattention/Hyperactivity

Inattention and hyperactivity were assessed using the parent-report version of the Strengths and Difficulties Questionnaire (SDQ) [25]. The SDQ is used to screen for emotional and behavioural problems in children aged 3–16 years. For the purpose of this study, only the five items comprising the inattention/hyperactivity subscale were used. These items capture inattention (2 items: “good attention span, sees chores or homework through to the end” and “easily distracted, concentration wanders”); hyperactivity (2 items: “restless, overactive” and “cannot sit still for long, constantly fidgeting or squirming”); and impulsivity (1 item: “thinks things out before acting”). Each item was scored on a 3 point scale *(not true = 0*, *somewhat true = 1*, *or certainly true = 2)*. Following the published scoring protocol, scores for each item were summed to compute a subscale score (range 0–10), with higher scores indicating greater problems. The SDQ inattention/hyperactivity subscale (parent proxy-report) has been shown to correlate highly with comparable items on the Child Behaviour Checklist (r = 0.71), and be at least as good as a semi-structured interview in detecting inattention/hyperactivity [26].

#### Classroom behaviour (fidgetiness, poor concentration, acting without thinking)

The SDQ was also used to identify parent perceptions of their child’s classroom behaviour using the following cross-informant question, *“Over the last 6 months has your child’s teacher complained of…*?*”* Parents reported their child’s classroom behaviour across 3 items including (a) fidgetiness, restlessness or overactivity; (b) poor concentration or being easily distracted; (c) acting without thinking, frequently butting in, or not waiting his or her turn. Response options were ‘no’, ‘a little’ and ‘a lot’. A scoring protocol was not provided for this item. As few parents responded with ‘a lot’, responses were dichotomised as ‘no’ and ‘a little/a lot’.

### Covariates

#### Socioeconomic position

The highest level of maternal education was reported through the parental questionnaire and provided a proxy measure of individual socioeconomic position (SEP). This was categorised as (1) mid to low SEP (below university education) and (2) high SEP (university education).

#### Physical activity

Waist worn ActiGraph GT1M accelerometers were used to provide an objective measure of children’s overall physical activity levels. The ActiGraph accelerometer is commonly used in studies involving children [27] and has documented evidence of validity and reliability for measuring children’s physical activity [28]. Children were asked to wear accelerometers during waking hours for eight consecutive days. Data were collected in 15-second epochs [29] and non-wear time was defined as ≥20 minutes of consecutive zeros [30, 31]. Freedson cut points were used to classify moderate- to vigorous-intensity physical activity [32]. Children’s overall physical activity was included in analyses if the accelerometer had been worn for a minimum of eight hours on at least four days, including one weekend day, expressed as average minutes/day of moderate- to vigorous- intensity physical activity (MVPA) [33], and adjusted for wear time [34]. MVPA was relatively normally distributed (skewness = 0.56; kurtosis = 3.21) and was only moderately correlated with duration of sports participation (r = 0.38;p<0.001).

### Analyses

Stata version 15 (StataCorp, Texas, USA) software was used for analyses. Independent samples *t*-tests were used to compare continuous variables, and chi square analyses were used to compare categorical variables between boys and girls. Associations between sport participation (duration and type) and classroom behaviour outcomes were explored using multiple linear regression for continuous outcomes (PedsQL mean school functioning and SDQ hyperactivity/inattention subscale scores) and multiple logistic regression for categorical outcomes (acting without thinking, fidgetiness and poor concentration). Two models were considered. In model 1, all analyses controlled for socioeconomic position and were adjusted for clustering by baseline centre of recruitment. Model 2 adjusted for overall physical activity in addition to socioeconomic position and clustering. Sex interactions were also explored by testing for interactions in the regression models, and stratifying by sex if significant interactions were observed. All analyses were completed on the whole sample and stratified by sex if significant sex interactions were observed. Only children with complete data were included for analyses.

## Results

The final analytic sample comprised of 438 boys and girls. Baseline characteristics of children in this sample were similar to those lost to follow up or excluded due to missing data in terms of sex (χ^2^ = 0.29;p = 0.59) but retained children were from higher maternal education backgrounds (χ^2^ = 22.63; p<0.001). Participant characteristics are shown in Table 1, along with sport participation over the past month, and scores for the three academic-related outcomes. The mean (standard deviation (SD)) age of participants was 10.56 (SD = 0.71) years. Eighty-nine percent of children participated in sport. Boys and girls spent a similar amount of time playing sport overall, however, more girls than boys participated in individual sport only, and more boys participated in team sport only. Further, the specific team and individual sports played by boys and girls differed. For team sports, girls most frequently participated in dance and netball while boys played soccer, basketball and football. For individual sports, girls tended to perform gymnastics, and boys tended to play tennis. Similar proportions of boys and girls participated in swimming, athletics and martial arts. For both boys and girls, duration of sport participation was associated with overall moderate- to vigorous-intensity physical activity (MVPA; p<0.001). MVPA was relatively normally distributed (skewness = 0.56; kurtosis = 3.21). Mean school functioning scores for boys and girls were slightly above the population norm of 76.91 [35]. Mean hyperactivity/inattention scores fell within the normal range (0–5) for the majority of boys and girls.

**Table 1:** Descriptive characteristics of participants.

	Total sample	Males	Females
**Demographic characteristics**			
Total sample, n (%)	438	239 (54)	199 (45)
Age in years, *mean*	10.56 (0.71)	10.58 (0.71)	10.53 (0.72)
Maternal education, n (%)			
< university	160 (37)	83 (35)	77 (39)
≥ university	278 (63)	156 (65)	122 (61)
**Duration of sport participation**			
Total hours per week, *mean*	4.02	4.22	3.78
**Type of sport participation; n (% yes)**			
Did not participate in sport last month	48 (11)	27 (11)	21 (11)
Participated in team and individual sport last month	168 (38)	92 (38)	76 (38)
Participated in individual sport only last month	104 (24)	46 (19)	58 (29)
Participated in team sport only last month	118 (38)	74 (31)	44 (22)
**Overall moderate- to vigorous-intensity physical activity**			
Total hours per day, *mean*	1.02	1.17	0.83
**Academic-related outcomes**			
School functioning score (range 0–100), *mean*	81.21	79.02	83.84
Hyperactivity score (range 0–10), *mean*	2.85	3.22	2.41
Classroom behaviour, n (%)			
Fidgetiness			
Never	379 (87)	193 (81)	186 (93)
At least sometimes	59 (13)	46 (19)	13 (7)
Poor concentration			
Never	306 (70)	143 (60)	163 (82)
At least sometimes	132 (30)	96 (40)	36 (18)
Acting without thinking			
Never	377 (86)	192 (80)	185 (93)
At least sometimes	61 (14)	47 (20)	14 (7)

Associations of (1) overall duration of sport participation and (2) sport type, with academic-related outcomes are shown in Table 2. All associations were in the hypothesised direction, although most were not significant. Weekly duration of sport participation was not associated with any outcomes. Results from model 1 show that compared with no sport, participation in individual sport was associated with scores 1.03 points lower for inattention/hyperactivity (individual sport: *B* = -1.03;95%CI:-1.73, -0.03). Further, compared with those who participated no sports, children who participated in individual sport had 65% lower odds of parent-reported acting without thinking at school. (OR = 0.35;95%CI:0.12,0.98). Children who participated in both team and individual sport scored 6.99 points higher for school functioning than children who participated in no sport (B = 6.99;95%CI:0.21,13.77). Only associations with individual sport remained significant in model 2, when MVPA was accounted for, (inattention/hyperactivity: *B* = -1.00;95%CI:-1.90, -0.00; acting without thinking: OR = 0.35;95%CI:0.13,0.98). Team sport participation was also associated with less inattention/hyperactivity (in model 2 only) (B = -0.88;95%CI:-1.73, -0.03). However, models accounted for only 2–3% of the variance in the classroom behaviour outcomes. There were no significant sex interactions.

**Table 2:** Associations between total duration of sport participation (hours/week), sport type and academic-related outcomes.

	School functioning score^1^ *B* (95% CI)		Inattention/hyperactivity^2^ *B* (95% CI)		Fidgetiness OR (95% CI)		Poor concentration OR (95% CI)		Acting without thinking OR (95% CI)	
TOTAL (n = 438)	Model 1	Model 2	Model 1	Model 2	Model 1	Model 2	Model 1	Model 2	Model 1	Model 2
Total duration of sport (hours/week)	0.22 (-0.38,0.82)	0.16 (-0.47,0.78)	0.04 (-0.05,0.12)	0.02 (-0.06,0.12)	0.94 (0.83,1.07)	0.94 (0.82,1.08)	1.04 (0.97,1.12)	1.03 (0.96,1.12)	1.00 (0.88,1.13)	0.99 (0.86,1.14)
Sport type^3^	F(3,118) = 1.53, p = .21	F(3,118) = 1.48, p = .22	F(3,118) = 1.69 p = 0.17	F(3,118) = 1.73 p = 0.16	χ^2^(3) = 6.75, p = .08	χ^2^(3) = 6.66 p = 0.08	χ^2^(3) = 4.97 p = 0.17	χ^2^(3) = 4.71 p = 0.19	χ^2^(3) = 4.83 p = 0.18	χ^2^(3) = 4.72 p = 0.19
Individual sport only	5.20 (-1.32,11.73)	5.32 (-1.24,11.88)	-1.03 (-1.93, -1.12)*	-1.00 (-1.90, -0.11)*	0.49 (0.16,1.47)	0.49 (0.16,1.48)	0.54 (0.24,1.21)	0.54 (0.24,1.22)	0.35 (0.12,0.98)*	0.35 (0.13,0.98)*
Team sport only	4.82 (-2.12,11.76)	4.51 (-2.40,11.42)	-0.82 (-1.68,0.04)	-0.88 (-1.73, -0.03)*	1.29 (0.43,3.84)	1.28 (0.43,3.79)	0.92 (0.44,1.92)	0.89 (0.43,1.86)	0.70 (0.27,1.79)	0.68 (0.27,1.75)
Both team and individual sport	6.99* (0.21,13.77)	6.77 (0.00,13.54)	-0.87 (-1.82,0.08)	-0.91 (-1.84,0.01)	0.78 (0.24,2.56)	0.78 (0.24,2.52)	0.59 (0.27,1.30)	0.57 (0.26,1.25)	0.59 (0.20,1.69)	0.58 (0.20,1.66)
MVPA	n/a	0.03 (-0.03,0.09)	n/a	0.01 (-0.00,0.01)	n/a	1.00 (0.99,1.01)	n/a	1.00 (0.99,1.01)	n/a	1.00 (0.99,1.01)
SEP	1.36 (-1.76,4.49)	1.24 (-1.91,4.38)	0.39 (-0.79,0.00)	-0.42 (-0.82, -0.02)*	0.97 (0.54,1.73)	0.97 (0.54,1.72)	0.65 (0.41,1.01)	0.64 (0.41,0.99)*	0.77 (0.39,1.53)	0.76 (0.38,1.51)

* denotes statistically significant associations (p<0.05)

^1^ Higher score = better school functioning

^2^ Higher score = more problems

^3^ Referent category is no sport

Model 1 –Multiple linear or logistic regression adjusted for SEP and clustering by centre of recruitment

Model 2 –Multiple linear or logistic regression adjusted for SEP, clustering by centre of recruitment and average minutes per day spent in moderate- to vigorous-intensity physical activity adjusted for wear time.

## Discussion

While studies show physical activity is positively associated with classroom behaviour outcomes, less is known about how different types of physical activity contribute to these outcomes. This study is one of the first to examine associations between sport participation and classroom behaviour outcomes among primary school-aged children and the first to consider this association within the context of overall physical activity. Results of this study suggest that while duration of weekly sport participation was not associated with classroom behaviour, participating in both team and individual sport was associated with enhanced school functioning, but not independent of overall physical activity. Results also showed that participation in individual sport was associated with reduced inattention/hyperactivity and acting without thinking, over and above the influence of physical activity.

The finding that children who participate in both team and individual sport have better school functioning is in line with results from a similar previous study among primary school-aged children [13]. However, that study did not adjust for overall physical activity. While not all sport participation involves moderate- to vigorous-intensity physical activity, [36, 37] in this study duration of sport participation was moderately correlated with overall moderate- to vigorous-intensity physical activity for both boys and girls. The current study found that associations were ameliorated, no longer reaching significance level, when physical activity was added to the model. This suggests that the associations for sport participation may merely be reflecting higher physical activity levels for those participating in more sport. However, the regression coefficient only reduced slightly with the addition of overall physical activity and both models explained only a small fraction of the variance in school functioning.

While the magnitude was modest, associations between participation in individual sport and both less inattention/hyperactivity and less acting without thinking were the most robust findings, being virtually unchanged after MVPA was accounted for. These findings suggests that participation in individual sport in particular may benefit classroom behaviour above and beyond that associated with increased MVPA. A possible explanation for this relates to the level of focus required when playing individual sports, compared with team sports; participants don’t have team mates to rely on thus any loss of focus will be more noticeable in individual sports [12]. This is one of the reasons individual sports are recommended for children with attention deficit hyperactivity disorder [12]. Pan and colleagues [11] reported that Taiwanese boys aged 6 to 12 years with attention deficit hyperactivity disorder displayed less parent reported attention problems following a 12-week table tennis (individual) intervention. As the current study was cross-sectional we do not know the direction of association, thus it could be that children who are more attentive play more individual sport. In contrast a study among kindergarten boys noted a link between participation in a team sport (soccer/football) program and improved attention [38]. Our study also reported team sport participation to be associated with less inattention, after accounting for MVPA. Similarly, another study reported that children who participated in team sport displayed better classroom engagement; no such association was seen for individual sport [14]. Those authors suggested that through participation in team sport, in particular, children may develop a unique sense of group belonging which may heighten the value they place on respecting rules and responsibilities, translating to better engagement in the classroom group setting [14]. However, with few studies and divergent results it is difficult to make solid conclusions.

While studies show there is a positive effect of MVPA on classroom behaviour [3], results of the current study do not support this relationship. In fact, results indicated that MVPA as measured by accelerometers was not associated with classroom behaviour outcomes. Most previous studies showing positive effects of physical activity on classroom behaviour have used self-report measures of physical activity [3], which are prone to social-desirability bias. Thus, a possible explanation for the null finding in the current study relates to reporter bias. Parent reports are used for both sports participation and classroom behaviours, while the accelerometer estimates of physical activity used in the current study are independent of parent-reported classroom behaviours.

Overall, results showed few associations between sport participation and academic-related outcomes. The finding that there were no sex differences in associations is unexpected given the evidence for sex differences in physical activity levels [39], sport participation [40], and academic-related outcomes [18]. However, the smaller sample size when dichotomised by sex may have contributed to a lack of findings.

### Strengths and limitations

The current study was one of the few to examine associations between sport participation and classroom behaviour or other academic-related outcomes among primary school-aged children. However, there are a number of limitations. Parent proxy-report of both sport participation and classroom behaviour outcomes introduce the potential for social desirability response bias. Further, parents may not be accurate reporters of their child’s behaviour at school given they are typically not present with their child during the school day. However, teacher and parent reports of child behavioural/emotional problems have been shown to be moderately correlated [41]. A further limitation of this study is its cross-sectional design, as causality between organised sport participation and academic-related outcomes could not be determined. Lastly, the inclusion of predominately classroom behaviour items limits the generalisability of findings to academic-related outcomes more broadly. Despite these limitations, the current study had several important strengths including comprehensive assessment of sport participation (any; type; duration), multiple measures of outcomes, and the inclusion of an objective measure of overall physical activity.

## Conclusions

The findings of this study indicate that sport participation, one form of physical activity, may be associated with less inattention/hyperactivity and parent reported acting without thinking, over and above the influence of physical activity. Parents may consider sport as one way to contribute to their child’s overall physical activity levels, although the impact of organised sport on classroom behaviour is modest at best.
